# Successful Treatment of Cancer with Arsenic

**Published:** 1885-04-20

**Authors:** J. McF. Gaston

**Affiliations:** Professor of Principles and Practice of Surgery in the Southern Medical College, Atlanta, Georgia


### Southern Medical Record.

EDITORS:

THOMAS S. POWELL, M. D. R. C. WORD, M. D.

*R. C. WORD, M.D., Managing Editor.*

☞All Communications and Letters on Business connected with the RECORD must be addressed to the Managing Editor.

VOL. XV. ATLANTA, GA., APRIL 20, 1885. No. 4.

### ORIGINAL AND SELECTED ARTICLES.

### SUCCESSFUL TREATMENT OF CANCER WITH ARSENIC.

By J. McF. GASTON, M. D.,
Professor of Principles and Practice of Surgery in the SOUTHERN MEDICAL COLLEGE, Atlanta, Georgia.

The ample provision made by two benefactors of suffering humanity for a cancer hospital in New York gives an importance at present to any measure of treatment for malignant tumors which affords a reasonable prospect of success. Two articles which appeared in the SOUTHERN MEDICAL RECORD of April 20th and of December the 20th in the past year, from Dr. H. M. Lawson, of Cuthbert, Ga., entitled, respectively, "On Malignant Diseases" and "Epithelioma," are entitled to special consideration as urging the arsenical medication of this class of diseases. In view of the evident fairness and good faith in the report of his salutary results, the medical profession should feel warranted in resorting to this agent for the correction of the true cancerous cachexy and the consequent modification of malignant tumors. Preliminary to entering upon the arsenical treatment, he insists upon using rhubarb as a laxative, and likewise in connection with it, if the arsenic does not act on the bowels. He states that one-eighth of a grain of arsenious acid will generally prove laxative; therefore, it is a good dose to begin with; but should it fail to impress the bowels, within three hours, a laxative dose of the rhubarb should be given, and repeated every three hours, until the bowels move. On the next morning, give one-fourth of a grain; and should this dose fail to prove laxative, pursue the same treatment with the rhubarb, until the laxative dose of arsenic is reached, when it is to be given every morning with a cup of hot coffee before breakfast.

The bowels becoming accustomed to that quantity, add one-eighth grain to the next dose given, and the laxative effect is to be maintained by adding the last-mentioned quantity as often as the case requires during the course of treatment. This augmentation may be safely continued until the dose reaches two grains, and few cases, if any, require a larger dose than one grain per day.

When the pain or other sensation ceases, a solution of the chloride of zinc, ten grains to the ounce of water, increased, if necessary, so as to produce a profuse discharge, will be found a serviceable resource. As soon as the morbid structures have melted down under the action of the caustic solution, he finds new, clean tallow superior to anything else as a dressing.

The arsenic should be continued in all cases until the parts are healed, and for a week afterward.

In withdrawing the remedy, especially if it has been found necessary to continue it longer than six weeks, or when it was necessary to increase it several times, it is not well to discontinue it too suddenly. Care must be taken to keep the bowels gently open during the entire time of withdrawing the remedy.

We are informed that the first dose of arsenic will be apt to produce nausea and, sometimes, vomiting, attended by slight pains in the stomach and griping pains in the bowels, somewhat like the effects of a drastic cathartic, but not so severe; but these effects are confined to the initial dose, the succeeding ones producing no unpleasant effects, but rather the contrary, nor is any further disturbance experienced unless it becomes necessary to increase the dose. All these unpleasant effects cease as soon as the bowels are moved, and are succeeded by a keen appetite and a feeling of excitement.

In view of the happy issue of a case in which there was lancinating pain and a peculiar sensation as of the gnawing and crawling of ants, after using arsenic for ten months, I was impressed with the efficacy of this mode of treatment, and determined that when an unmistakable case of malignant disease should present itself I would test these herculean doses of arsenic.

Accordingly, there appeared at my clinic on the 31st Dec., 1884, a man with a well-defined encephaloid tumor, having all the characteiistics of fungus hæmatodes, located immediately beneath the right ear, with its anterior border encroaching upon the angle of jaw and the posterior margin covering the mastoid process, while it descended upon the sterno-cleido mastoid muscle.

The patient exhibited the cancerous cachexy most emphatically, and the diagnosis was further corroborated by the circumstance that an operation had previously been performed for the removal of the tumor, with its prompt reproduction in the same locality.

After drawing the attention of the class of the Southern Medical College to the cauliflower protuberance on all sides of the elevated growth that exuded a bloody serum, I prescribed 5 drop doses of Fowler's arsenical solution three times a day, with instructions to add a drop every alternate day until he should reach ten drops of the solution three times a day.

After using the remedy in this form for nearly a month, I changed it for the arsenious acid in the dose of ¼ grain daily with a cup of hot coffee before his morning meal, and so soon as tolerance of this was apparent a second dose was given before dinner, and ultimately he took a third dose before supper, with indications of improvement in all respects. About the first of March the dose was increased to ⅓ grain of arsenious acid three times a day, and continued for ten days, when he manifested signs of constitutional disturbance, with some puffing under the eyes and tenderness over the epigastrium, with evident modification in the character of the tumor. All the indications of a malignant growth having disappeared, and a marked diminution in its proportions being evident, while the raw surface had shrunk down to the level of the surrounding integument, there were still exuberant granulations accompanied with a purulent discharge. Up to this time a simple aseptic local dressing of vaseline ℥ j, with carbolic acid and camphor, each ℨ i, had been applied with absorbent cotton; but then I prescribed vaseline ℥ j, with chloride of zinc 30 grs., on a piece of cloth upon the raw surface daily. At this period, on the 10th of March, one-third of the arsenious acid was abstracted, leaving him to take ⅓ grain before his breakfast and a like quantity before supper. At the expiration of another ten days he was ordered to stop the evening portion, and has since continued taking only one-third of a grain before breakfast. The acceleration of the pulse, with the slight signs of bloating about the face, have disappeared under this reduction of the daily dose, and his appetite, which had become somewhat impaired, is now being restored, while the tenderness and distention of the epigastrium gives no further inconvenience.

The exuberant granulations having been repressed by the local application of the chloride of zinc, it has been discontinued, and a simple plaster of basilicon ointment on a cloth covers the granulating surface. It appears, not only to myself, but to various colleagues who have observed the progress of this case under the arsenical course, that while a specific taint remained in his system there was a tolerance of the arsenic, but as soon as there was a marked change in the general and local indications of disease for the better, the peculiar effects of this agent become more developed. We may reason from the analogy afforded in the tolerance of iodide of potash by subjects of syphilis that a similar diminished susceptibility to the effects of arsenic is conferred by the existence of malignant disease, and that, consequently, when it is combatted the influence of the arsenic should be more shown, as in this case, upon the evidence of a decline in the specific element.

Frequent evacuations from the bowels accompanied the other evidences of arsenical disturbance during the latter period of the full dose of 1 grain per day, and this has also ceased with the reduction of the quantity to $\frac{1}{3}$ grain a day. It is my intention to fall back to $\frac{1}{4}$ grain of arsenious acid from the present, and within ten days to reduce it to $\frac{1}{8}$ grain once a day.

The improvement is so marked, and so fully recognized by other members of the medical profession who have watched the various stages of the case, that it should encourage further use of this remedy, and I have been prompted by the hope of final success to lay the history of the progress thus far before those who may have an opportunity to test the virtues of the arsenical treatment for the various forms of malignant disease.

It will be observed that I have not adhered to the plan of Dr. Lawson, in the administration of divided doses instead of giving all that was intended for the day in one portion; and that the chloride of zinc has been applied as a paste with vaseline, instead of a watery solution that would require to be more frequently repeated and be liable to run off upon the sound skin. Others will doubtless consider the advantages of distributing the influence of the arsenic throughout the twenty-four hours rather than obtaining the purgative effect at a single hour in the morning, but the laxative tendency was evident even when it was thus given at three different times in the day.

A circumstance connected with the management of this case impresses me with the importance of keeping the patient in ignorance of the fact that arsenic is taken. Being presented on a second occasion before the class, I dwelt upon the properties of this agent, and he became aware that more energetic effects were expected than are ordinarily obtained, though he was taking then less than 10 drops of Fowler's solution three times a day. From this time he manifested concern about the influence of the remedy on his organization, and I resorted to the trick of changing his medicine from a fluid to a powder without letting him know that it was arsenic. There was no further apprehension and no further complaint of trouble until the maximum dose of $\frac{1}{3}$ grain of arsenious acid three times was continued for some days; and thus one grain, corresponding to two fluid drachms of Fowler's solution, in the twenty-four hours was taken, not only with impunity, but with evident good result in arresting the disease.

A further report, giving the final outcome of this treatment, will be presented at a future day.

